# Optimization and scale-up of cell culture and purification processes for production of an adenovirus-based tuberculosis vaccine

**DOI:** 10.1186/1753-6561-9-S9-P23

**Published:** 2015-12-14

**Authors:** Chun F Shen, Danielle Jacob, Zhongqi Shao, Alice Bernier, Xuefeng Yu, Mehul Patel, Tao Zhu, Amine Kamen

**Affiliations:** 1 Vaccine Program, Human Health Therapeutics Portfolio, National Research Council of Canada, Montreal, H4P 2R2, Canada; 2 Tianjin CanSino Biotechnology Inc., Tianjin, China; 3 Department of Bioengineering, Macdonald Engineering Building, McGill University, Montréal, Québec, H3A 0G4, Canada

## Background

Tuberculosis (TB) is the second leading cause of death by infectious disease worldwide. About 1.4 million people die of TB each year. Parenterally administered Mycobacterium bovis BCG vaccine confers only limited immune protection from pulmonary tuberculosis in humans. There is a need for developing effective boosting vaccination strategies. AdAg85A adenovirus, a new promising tuberculosis vaccine candidate, has been studied with mouse, guinea pig, goat and cow animal models, and was shown to be effective against Mycobacterium tuberculosis (Mtb) infection [1]. A phase I trial on Ad5Ag85A adenovirus was also conducted, which demonstrated that AdAg85A adenovirus was safe and highly immunogenic [2].

To further evaluate the efficacy of this vaccine and reduce the cost of this promising vaccine candidate, a feasible and cost-effective large-scale cell culture production process had to be developed for manufacturing large quantities of AdAg85A adenovirus required for further clinical trials. Furthermore, the process had to be designed to meet all requirements for industrialization and commercialization of this vaccine candidate. Here we report our study on optimization of cell culture conditions, scale up of AdAg85A adenovirus production in 60L bioreactor and purification of the AdAg85A adenovirus at different scales. The optimized conditions for AdAg85A adenovirus production and purification were transferred to a GMP facility for manufacturing of AdAg85A adenovirus for further clinical trials.

## Materials and methods

Four commercial serum-free cell culture media (SFM4HEK-293 and SFM4Transfx-293 from HyClone; Adenovirus Expression Medium (AEM) and CD 293 from Life Technologies), were evaluated for supporting the growth of HEK293SF-3F6 cell in suspension and also for the production of AdAg85a adenovirus in 125 mL shake flask cultures under various experimental conditions. The production of the AdAg85A adenovirus was then scaled up to 3L controlled bioreactor under the optimized conditions obtained from the shake flask experiment, further validated in a 60L bioreactor.

Purification of the AdAg85A adenovirus was accomplished through many different steps. Some of the critical steps include cell lysis, benzonase® treatment, Q-Sepharose HP anion exchange chromatography for capture of adenovirus/purification, Capto Core 700 multimodal chromatography for polishing, concentration and diafiltration into formulation buffer. The purification processes were also scaled up from 3L to 60 L production scale.

## Results

**Figure 1:**
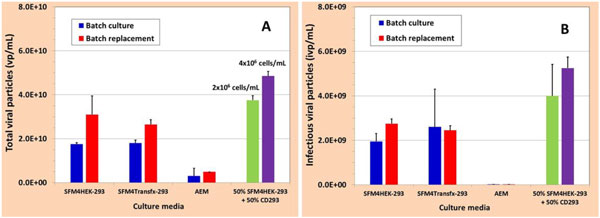
**Screening of culture media and optimization of culture conditions for adenovirus production (A: total viral particle concentration; B: infectious viral particle concentration)**. The cells grown in the above 3 media were infected at 1x10^6 ^cells/mL with or without medium replacement before infection.

Three among four media supported growth of HEK293SF-3F6up to 4x106 cells/mL, however, the production yield of AdAg85A in the three media varied considerably. More than one log difference in viral titres has been observed as shown in Figure 1.Although CD293 medium did not support the cell growth, however, the virus production was further improved by more than two folds when the cells were grown in SFM4HEK293 medium to respective cell density of 2x106 cells/mL and 4x106 cells/mL, and then diluted with the same amount of CD 293 medium before the virus infection. This result clearly indicates that HEK293 has different nutritional requirements during the phases of cell growth and virus production. Highest titer (5x1010 total viral particles/mL) of AdAg85A adenovirus was achieved by growing HEK293SF-3F6 cell in one of the above 3 media to a cell density of about 4x106 cells/mL, and then diluting the culture with the same of CD293 before the virus infection. These optimized culture conditions eliminated the medium exchange step and contamination risk associated with, and also contributed to reducing the cost of goods.

The production of AdAg85A adenovirus was successfully scaled up to 3L bioreactor, and then validated in 60L bioreactor production. Experimental result showed that configuration of bioreactor might significantly affect the yield of AdAg85A adenovirus

Purification of AdAg85A adenovirus was successfully scaled up to 60L scale production. Experimental data revealed that an additional step of freeze-thaw following the cell lysis helped the release of AdAg85A adenovirus from the cell and thus improved the virus recovery. Recovery rate of total viral particles was more than 60% after anion exchange and Capto Core 700 multimodal chromatography for polishingstep (Table 1). However, experimental result also showed that it was a challenge to use a stir cell system to further concentrate the flow through (with 2x1012 viral particles/mL) from Capto Core 700 multimodal chromatography step.

**Table 1:** Recovery of total and infectious viral particles and removal of total proteins in purification steps in a 3 L scale run.

Samples	Total viral particles	Recovery	Total infectious particles	Recovery	Total proteins (mg)	Recovery
Harvest broth	7.1 × 10^13^	100%	6.5 × 10^12^	100%	1112	100%
10X conc. lysate	5.1 × 10^13^	72%	5.7 × 10^12^	88%	993	89%
Q-Seph HP feed	7.2 × 10^13^	101%	5.0 × 10^12^	77%	882	79%
Q-Seph HP 1M NaCl peak	5.3 × 10^13^	75%	3.8 × 10^12^	59%	68	6%
Capto Core 700 flowthrough	4.3 × 10^13^	61%	3.2 × 10^12^	49%	11	1%
Final product	2.0 × 10^13^	28%	2.6 × 10^12^	40%	4	0.4%

More than 99% of host cell protein was removed during the purification process. Total protein concentration was 260 µg/mL in the purified product. Purity of the purified product was equal to that of the ATCC VR-1516 Ad5 standard by SDS-Page, silver stain analysis. The ratio of infectious viral particles to total viral particles in the purified material was in a range of 10%. Endotoxin concentration of the purified material was lower than 0.10 EU/mL.

## Conclusions

A high yield bioprocess was successfully developed, and scaled up to 60L scale for production of AdAg85A adenovirus using HEK293SF 3F6 cell and serum-free media. The developed downstream process efficiently purified the AdAg85A produced at different scales and with acceptable virus recovery.
